# A randomised controlled trial to investigate the clinical effectiveness and cost effectiveness of Mindfulness-Based Cognitive Therapy (MBCT) for depressed non-responders to Increasing Access to Psychological Therapies (IAPT) high-intensity therapies: study protocol

**DOI:** 10.1186/s13063-022-06882-w

**Published:** 2023-01-19

**Authors:** Thorsten Barnhofer, Barnaby D. Dunn, Clara Strauss, Florian Ruths, Barbara Barrett, Mary Ryan, Asha Ladwa, Frances Stafford, Roberta Fichera, Hannah Baber, Ailis McGuinness, Isabella Metcalfe, Delilah Harding, Sarah Walker, Poushali Ganguli, Shelley Rhodes, Allan Young, Fiona Warren

**Affiliations:** 1 School of Psychology, University of Surrey, Guildford, UK; 2 Department of Psychology, University of Exeter, Exeter, UK; 3 University of Sussex, Brighton, UK; 4 South London and Maudsley NHS Foundation Trust, London, UK; 5 King’s Health Economics, King’s College London, London, UK; 6 Department of Health and Social Care Innovation Lab, Southbank University, London, UK; 7 College of Medicine and Health, University of Exeter, Exeter, UK; 8 Centre for Affective Disorders, King’s College London, London, UK

**Keywords:** Mindfulness-Based Cognitive Therapy, Major depressive disorder, Treatment non-response, Increasing Access for Psychological Therapies (IAPT)

## Abstract

**Background:**

Major depression represents a pressing challenge for health care. In England, Increasing Access to Psychological Therapies (IAPT) services provide evidence-based psychological therapies in a stepped-care approach to patients with depression. While introduction of these services has successfully increased access to therapy, estimates suggest that about 50% of depressed patients who have come to the end of the IAPT pathway still show significant levels of symptoms. This study will investigate whether Mindfulness-Based Cognitive Therapy (MBCT), a group intervention combining training in mindfulness meditation and elements from cognitive therapy, can have beneficial effects in depressed patients who have not responded to high-intensity therapy in IAPT. It will seek to establish the effectiveness and cost-effectiveness of MBCT as compared to the treatment these patients would usually receive.

**Methods:**

In a 2-arm randomised controlled trial, patients who currently meet the criteria for major depressive disorder and who have not sufficiently responded to at least 12 sessions of IAPT high-intensity therapy will be allocated, at a ratio of 1:1, to receive either MBCT (in addition to treatment as usual [TAU]) or continue with TAU only. Assessments will take place at baseline, 10 weeks and 34 weeks post-randomisation. The primary outcome will be reduction in depression symptomatology 34 weeks post-randomisation as assessed using the Public Health Questionnaire-9 (PHQ-9). Secondary outcomes will include depressive symptomatology at 10 weeks post-randomisation and other clinical outcomes measured at 10-week and 34-week follow-up, along with a series of binarised outcomes to indicate clinically significant and reliable change. Evaluations of cost-effectiveness will be based on assessments of service use costs collected using the Adult Service Use Schedule and health utilities derived from the EQ-5D.

**Discussion:**

This trial will add to the evidence base for the use of MBCT in depressed treatment non-responders. It will constitute the first trial to test MBCT following non-response to psychological therapy, with results providing a direct estimate of efficacy within the IAPT pathway. As such, its results will offer an important basis for decisions regarding the adoption of MBCT for non-responders within IAPT.

**Trial registration:**

ClinicalTrials.gov NCT05236959. Registered on 11 February 2022. ISRCTN 17755571. Registered on 2 February 2021.

**Supplementary Information:**

The online version contains supplementary material available at 10.1186/s13063-022-06882-w.

## Administrative information

Note: the numbers in curly brackets in this protocol refer to SPIRIT checklist item numbers. The order of the items has been modified to group similar items (see http://www.equator-network.org/reporting-guidelines/spirit-2013-statement-defining-standard-protocol-items-for-clinical-trials/).

**Table:** 

Title {1}	A randomised controlled trial to investigate the clinical effectiveness and cost-effectiveness of Mindfulness-Based Cognitive Therapy (MBCT) for depressed non-responders to Increasing Access to Psychological Therapies (IAPT) high-intensity therapies
Trial registration {2a and 2b}.	ClinicalTrials.gov: NCT05236959, 11.02.2022 ISRCTN 17755571, 02.02.2021
Protocol version {3}	Version 3.0 [28.10.2022]
Funding {4}	This trial is funded through the Research for Patient Benefit (RfPB) Programme of the National Institute for Health Research (NIHR). The funders number for this trial is NIHR200750.
Author details {5a}	Thorsten Barnhofer, University of Surrey, School of Psychology, Guildford, UK GU2 7XH, phone: 01,483 686,485, email: t.barnhofer@surrey.ac.uk Barney Dunn, University of Exeter, Department of Psychology, Washington Singer Laboratories, University of Exeter, Perry Road, Prince of Wales Road, Exeter, EX4 4QG, email: b.d.dunn@exeter.ac.uk Clara Strauss, University of Sussex, Sussex House, Falmer, Brighton, BN1 9RH, United Kingdom, email:c.y.strauss@sussex.ac.uk Florian Ruths, South London and Maudsley NHS Foundation Trust, IPTT Southwark, Maudsley Hospital Outpatient Building, 105 Denmark Hill, London SE5 8AZ, email: florian.ruths@slam.nhs.uk Barbara Barrett, King’s College London, King’s Health Economics, Box P024, Institute of Psychiatry, Psychology & Neuroscience, De Crespigny Park, London, SE5 8AF, email: barbara.m.barrett@kcl.ac.uk Mary Ryan, Department of Health and Social Care Innovation, London South Bank University, 103 Borough Road, SE1 0AA, email: mary@highbag.co.uk Asha Ladwa, Devon Partnership Trust, Wonford House, Dryden Road, Exeter, EX2 5AF, email: asha.ladwa@nhs.net Frances Stafford, Sussex Partnership NHS Foundation Trust, Hove, BN3 7HY, email: frances.stafford@spft.nhs.uk Roberta Fichera, South London and Maudsley NHS Foundation Trust, IPTT Southwark, Maudsley Hospital Outpatient Building, 105 Denmark Hill, London SE5 8AZ, email: roberta.fichera@slam.nhs.uk Hannah Baber, Devon Partnership Trust, Wonford House, Dryden Road, Exeter, EX2 5AF, email: hannah.baber@nhs.net Ailis McGuinness, University of Exeter, Department of Psychology, Washington Singer Laboratories, University of Exeter, Perry Road, Prince of Wales Road, Exeter, EX4 4QG, email: a.mcguinness@exeter.ac.uk Isabella Metcalfe, South London and Maudsley NHS Foundation Trust, IPTT Southwark, Maudsley Hospital Outpatient Building, 105 Denmark Hill, London SE5 8AZ, email: isabella.metcalfe@slam.nhs.uk Delilah Harding, South London and Maudsley NHS Foundation Trust, IPTT Southwark, Maudsley Hospital Outpatient Building, 105 Denmark Hill, London SE5 8AZ, email: delilah.harding@nihr.ac.uk Sarah Walker, University of Exeter, College of Medicine and Health, Smeal Building, St Luke’s Campus, Heavitree Road, Exeter, EX1 2LU, email: s.walker@exeter.ac.uk Poushali Ganguli, King’s College London, King’s Health Economics, Box P024, Institute of Psychiatry, Psychology & Neuroscience, De Crespigny Park, London, SE5 8AF, email: poushali.ganguli@kcl.ac.uk Dr Shelley Rhodes, University of Exeter, College of Medicine and Health, College House, St Luke’s Campus, Heavitree Road, Exeter, EX1 2LU, email: s.rhodes@exeter.ac.uk Prof Allan Young, King’s College London, Centre for Affective Disorders, Institute of Psychiatry, Psychology & Neuroscience, King’s College London, PO72 De Crespigny Park, London SE5 8AF, United Kingdom, phone: 020 78,480,086, email: allan.young@kcl.ac.uk Dr Fiona Warren, University of Exeter, College of Medicine and Health, Smeal Building, St Luke’s Campus, Heavitree Road, Exeter, EX1 2LU, phone: 01,392 722,749, email: f.c.warren@exeter.ac.uk
Name and contact information for the trial sponsor {5b}	Sussex Partnership NHS Foundation Trust, Ms Taffy Bakasa, Lead Governance Officer, R & D Department, Sussex Education Centre, Nevill Avenue, Hove, BN3 7HY, Tel: 0300 3,040,088, Email: taffy.bakasa@sussexpartnership.nhs.uk
Role of sponsor {5c}	The trial sponsor has ultimate authority over the management of the study. Neither the funder nor the sponsor of the trial was involved in the design of the study and will not be involved in the collection, analysis or interpretation of data or the writing of the study report. The funder will be required to approve the final report prior to publication.

## Introduction

### Background and rationale {6a}

Major depression represents a pressing challenge for health care. The disorder not only is highly prevalent—10.9% of the adult population in England suffered from an episode of depression in 2014 [1]—but also shows many characteristics of a progressive disease: if left untreated, it tends to become more recurrent and chronic over time [2], with even residual levels of symptoms conferring a significantly increased risk for future relapse [3]. There is evidence for functional decline as the disorder accelerates [4], and physiological changes underlying its progression have been linked with a significantly increased risk for a broad range of physical and neurodegenerative disorders [5].

In order to address this challenge, it is imperative to provide treatments that effectively reduce symptoms in those who are affected and promote recovery and thus to prevent progression into increasingly recurrent or chronic courses. Although there is still a significant unmet need, progress in providing access to treatment has been encouraging [1], due to a significant degree to the introduction of Improving Access to Psychological Therapies (IAPT) services, which were established with the express aim of providing patients with evidence-based psychological treatments in a timely manner and are projected to care for increasing numbers of patients [6]. IAPT uses a stepped care approach, with those not responding to low-intensity treatment or greater complexity receiving high-intensity treatment. In 2020–21, IAPT services offered treatment to over 1,000,000 people.

However, while the introduction of IAPT is successfully increasing access to psychological therapies, outcome reports indicate that about 50% of the depressed patients who have completed high-intensity evidence-based psychological therapies within IAPT do not reach recovery and continue to show significant levels of symptoms. [7]. At the same time, progression to secondary care remains reserved for those with complex depression and high risk for suicidality. Data from the “Predicting Outcome Following Psychological Therapy in IAPT (PROMPT)” study show that of those who do not respond only 8% receive secondary care interventions [8, 9], while the remainder are currently not offered any further-line treatment. Most of these patients are sent back to their GPs, who are likely to prescribe antidepressant medication. Yet, the majority of IAPT non-responders are already receiving medications [8, 9]. There is therefore a considerable gap in service provision for patients who do not respond sufficiently to high-intensity evidence-based psychological therapies—a problem that is likely to come into focus even further as numbers of patients accessing IAPT are increasing.

Mindfulness-Based Cognitive Therapy (MBCT) [10], an 8-week, group-based intervention that combines intensive training in mindfulness and elements from cognitive therapy for depression, may be particularly suited for addressing this gap. While originally developed, and NICE-recommended, for the prevention of relapse in remitted patients with a history of recurrent depression, recent research has brought promising evidence that MBCT can have significant beneficial effects in patients with acute and more persistent forms of the disorder [11, 12], and particularly in those who have not responded to previous interventions [13]. In a large definitive RCT in patients who had not responded to antidepressants, Eisendrath et al. [13] found a statistically significant advantage of MBCT over a rigorous psychological control treatment on depression symptomatology, *d* = 0.32, an effect size in the small to medium range. In a smaller scale RCT, also in patients who had not responded to antidepressants, Chiesa et al. [14] reported a statistically significant benefit for MBCT relative to attention-placebo on depression symptomatology, *d* = 0.79, an effect size in the medium to large range. A further smaller scale RCT investigating the effects of MBCT in chronically depressed patients who had not responded to antidepressants has shown a statistically significant advantage on depressive symptomatology compared with treatment as usual with a small effect size (*d* = 0.35) [15]. However, evidence is currently not sufficient to warrant guideline endorsement for use as a further-line treatment, which is a necessary prerequisite for implementation within the evidence-based IAPT pathway.

The current trial will constitute a second definitive trial of MBCT as a further-line treatment and would thus provide an important step towards a sufficient evidence base. Furthermore, it will constitute the first trial to test MBCT following non-response to psychological therapy with results providing a direct estimate of efficacy within the IAPT pathway. If successful, the proposed research would provide the evidence necessary for adoption of MBCT for non-responders within IAPT and would thus justify the use of an easy to implement and much-needed treatment option for a considerable proportion of patients who are currently not receiving sufficient support.

MBCT offers a number of advantages for addressing more persistent courses of depression. Mindfulness training is specifically aimed at helping patients become better at recognising and disengaging from habitual and automatic maladaptive patterns of thinking. Research indicates that, through such ‘decentering’, the practice helps to prevent the spiralling of negative mood [16]. The training provides patients with sustainable skills that remain accessible to them after the end of the intervention, with recent evidence suggesting that ‘decentering’ skills further improve as patients continue using mindfulness practices following the end of the intervention [17]. MBCT might thus serve to effectively reduce symptoms [11, 12] as well as keep people well for the longer term [18]. Because of its group-based format and emphasis on training skills, the intervention is particularly suited for alternative forms of delivery such as videoconferencing.

In order to provide evidence that, if positive, would be sufficient to enable a change in IAPT practice, we shall compare MBCT (in addition to TAU) as delivered via videoconferencing to treatment-as-usual (TAU) in IAPT high-intensity treatment non-responders in a definitive clinical trial. TAU was chosen as the comparator as it is reflective of the current state of care. Delivery via videoconferencing was chosen due to demands of the COVID-19 pandemic. We shall test the immediate effects of the intervention on depressive symptomatology as well as whether effects on symptomatology can be sustained over a period of 6 months (the primary outcome), thus taking into account the high risk of relapse in early stages following treatment. In addition to testing clinical effectiveness, we shall measure service use and collect information on quality of life in order to provide information on the cost-effectiveness of the intervention in IAPT high-intensity treatment non-responders. Previous research has suggested that MBCT for relapse prevention is broadly comparable to maintenance antidepressant use with an estimated cost for group attendance in person of £112 per group participant [19] with these costs likely to be lower when delivering the intervention via remote formats such as videoconferencing. However, data on the economic effects of outcome in IAPT non-responders would be needed in order to guide decisions on implementation in this particular group.

## Objectives {7}

### Aims

To establish (a) the clinical effectiveness (in terms of reductions in depressive symptomatology) and (b) cost-effectiveness of MBCT as a psychotherapeutic treatment option for depressed patients who have not responded sufficiently to high-intensity evidence-based treatments within the IAPT care pathway compared with TAU.

### Objectives

- To undertake a definitive randomised controlled trial (RCT) of the MBCT intervention versus TAU to confirm clinical effectiveness of the treatment in depressed non-responders to high-intensity evidence-based treatments within the IAPT care pathway, and
- To use the data from the RCT to conduct a cost-utility and cost-effectiveness analysis to provide information on whether or not the MBCT intervention is worthwhile economically.

### Hypotheses

We hypothesise:

- That participants who receive MBCT will show significantly stronger reductions in depressive symptomatology than participants who receive TAU both at 10 weeks post-randomisation (post-treatment; secondary outcome) and at 34 weeks post-randomisation (primary outcome).
- That the MBCT intervention will be cost-effective either in terms of reductions in costs elsewhere in the health system or in improvements in outcomes at 34 weeks post-randomisation.

We will also investigate effects on a range of other secondary outcomes including measures of co-morbid anxiety symptoms, social functioning and quality of life as well as potential process variables including decentring and mindfulness (see further below).

Qualitative analyses will investigate acceptability and implementability of MBCT taking into account the particular type of delivery format chosen.

## Trial design {8}

This is a two-arm parallel group randomised controlled superiority trial. We will randomly allocate 234 patients who have not responded to high-intensity IAPT interventions for depression, but do not meet eligibility criteria for secondary care services, to receive MBCT or to continue with TAU, providing a comparator that is reflective of the current state of care (and in most cases will entail continued use of antidepressant medication). We will measure outcomes at baseline, 10-week and 34-week follow-up post-randomisation. Economic analyses will investigate effects of the interventions on subsequent service use and health-related quality of life.

## Methods: participants, interventions and outcomes

### Study setting {9}

The study will be conducted at three research sites in the UK: at the Sussex Mindfulness Centre, Sussex Partnership Trust, where we will be working in collaboration with the University of Surrey; the Mood Disorders Centre at the University of Exeter; and the Centre for Affective Disorders at King’s College London, Institute of Psychiatry, Psychology & Neuroscience. Assessments will be conducted remotely, using videoconferencing, telephone and links to web-based questionnaires, by researchers at the three research sites. Data management will be provided by the Exeter Clinical Trials Unit at the University of Exeter. Treatments will be delivered via videoconferencing by therapists at the Centre for Affective Disorders, King’s College, London, where we will be working in collaboration with the Maudsley Mindfulness Service and South London and Maudsley (SLaM) IAPT services, at Sussex Mindfulness Centre, where we will be working in collaboration with Sussex Partnership Foundation Trust and Sussex Community Trust IAPT services, and at the AccEPT Clinic, Mood Disorders Centre, University of Exeter, where will be working in collaboration with the Devon Partnership Trust IAPT service. The research sites will include further patient identification centres (PICs) where needed and helpful. Potential PICs will need to be able to recruit a considerable number of patients and in terms of their organisational features should not show outlier characteristics (for more detail see Sect. 15. Recruitment).

### Eligibility criteria {10}

We will recruit depressed treatment non-responders to IAPT high-intensity treatments into the study. 

*Inclusion criteria* will be:

1. Non-response to a minimal effective dose of high-intensity treatment for depression (primary presenting problem) in IAPT (at least 12 sessions, in line with NICE guideline suggestions) defined in line with the caseness threshold adopted by IAPT as a Patient Health Questionnaire-9 (PHQ-9) [20] score of 10 or higher.
2. Meeting criteria for a current episode of major depression according to DSM-5 as assessed through the Mini International Neuropsychiatric Interview for DSM-5 (MINI 7.0.2) [21] along with a current PHQ-9 score of 10 or higher.
3. Age 18 or older.
4. Access to a working Internet connection and equipment to participate in videoconferencing assessments and interventions.

According to the IAPT database, the majority of patients who receive high-intensity psychological treatment will also have received treatment with antidepressant medication, and most of these patients will therefore meet consensus criteria for treatment resistance. We will compare the sociodemographic characteristics of our sample against the characteristics of the wider group of people attending the collaborating IAPT services in order to judge the representativeness of the sample.

Potential participants will be *excluded* if

1. Based on the judgement of their IAPT therapist they are eligible for, would be seen by, and their needs would be best met by secondary care specialist services.
2. They present with a level of risk to self or others that cannot be safely managed in a primary care service context (i.e. active suicidal plans), a history of psychosis or psychotic symptoms, a current episode of mania, alcohol or substance abuse or dependence within the past 3 months, a current post-traumatic stress disorder, an obsessive–compulsive disorder or an eating disorder.
3. They suffer from any other significant disease or disorder that may either put the participant at risk because of participation in the trial, or may influence the result of the trial, or the participant’s ability to participate in the trial.
4. They have an insufficient ability to understand or read English.

Patients who are currently taking antidepressant medication will be allowed into the trial and medication use will be documented for statistical analysis. Medication use will be included as a minimisation variable in the randomisation procedure.

### Who will take informed consent? {26a}

Informed consent will be obtained in a two-phase consent process. Participants will receive a study summary sheet, produced in line with current Health Research Authority (HRA) guidelines (http://www.hra-decisiontools.org.uk/consent/index.html) and informed by patient representatives, via email through the service and first give permission for the research team to contact them for discussion of the study and screening in an initial call. Full informed consent will be taken in person by a study researcher prior to the eligibility and baseline assessment to be conducted via videoconferencing. Potential participants will receive full information about the study in advance of the interview. At the point of consent, there will be further opportunity to discuss the study and for the participant to raise any questions. The opportunity to withdraw from the trial will be fully explained. Researchers will be trained in taking informed consent, including assessment of capacity to consent where appropriate, and supervised by the CI and site leads. Consent will be taken only from individuals with capacity to make an informed decision on their participation.

### Additional consent provisions for collection and use of participant data and biological specimens {26b}

On the consent form, participants will be asked if they agree to be contacted about ethically approved research studies for which they might be suitable. Participants will also be asked, if they agree to their anonymised data, anonymised transcripts, and session recordings being used in future research. For more details, see the copy of the consent form at the end of this article. This trial does not involve collecting biological specimens.

### Interventions

#### Explanation for the choice of comparators {6b}

TAU was chosen as comparator as it is reflective of the current state of care for patients who have not responded to high-intensity therapy in IAPT.

#### Intervention description {11a}

MBCT combines mindfulness training with elements from cognitive therapy. We will follow the treatment manual with minor adaptations to address the fact that patients are suffering from current symptoms of depression following practice from our previous research [11, 12]. The intervention will be delivered by trained MBCT therapists together with an assistant to groups with a target size of 13 patients (minimum 8 and maximum of 16) using videoconferencing on a secure online platform. This will allow participants to attend sessions through Internet connection from their home or another place of their choosing. All three sites have prior experience with delivering MBCT in this format and will follow shared internal guidelines for videoconferencing delivery. All therapists will meet qualifications in line with Good Practice Guidelines and competency level ‘proficient’ on the MBCT Therapy Pathway, which implies, among other criteria, that the therapist is teaching at competent levels across all six domains of the Mindfulness-Based Interventions: Teaching Assessment Criteria (MBI-TAC, [22]). Where MBI-TAC ratings are not available, we will require the therapist to have a track record of teaching MBCT for at least 5 years. Therapists will receive a 1-day workshop to familiarise them with the modifications of the programme necessary for use with currently depressed patients and will be provided with individual supervision sessions weekly during the intervention. Manual adherence and treatment fidelity will be monitored using methods established in our previous trials using the MBCT Adherence Scale [23] and MBI-TAC based on the recordings of the online intervention sessions. MBCT consists of eight weekly group-based sessions and participants are asked to engage in home practice for about an hour per day using guided meditation audio recordings, with attendance and practice monitored following previously established practices [10]. As the intervention is delivered online, it will be possible for participants recruited at different sites to attend a given MBCT course. We will offer access to an online MBCT course run by therapists at a centre different from the one where the participant has been recruited, if it is deemed helpful in order to respond to demands of recruitment and time preferences by participants and provided that risk management procedures remain unaffected. In these cases, assessments will continue to be conducted by the site where the participant has been recruited and we will require information about local emergency contacts to be in place and provided to the therapist of the group.

Participants in the TAU condition will be asked to continue with their usual care and follow the regimens suggested by their GP or mental health professional, which in most cases will consist of continuing use of antidepressant medication. Following previous practice in our trials [18], TAU participants will be invited to an interview to prevent tendencies towards ‘resentful demoralisation' and highlight the importance of their contribution. As the pre-class interview for the MBCT courses, this interview will be conducted via videoconferencing.

#### Criteria for discontinuing or modifying allocated interventions {11b}

Participants are free to withdraw their participation at any point. If a participant in either arm indicates that they wish to discontinue the trial they will not be contacted further by the research team, other than to invite them to take part in a brief written survey to ascertain their reasons for not taking part. In the MBCT arm of the trial, different levels of discontinuation are possible. A participant may discontinue therapy but remain in the trial, or they may discontinue the trial. In order to enable intention-to-treat analyses, we will still ask participants who opt to discontinue therapy at any point to take part in assessments, should they be willing to contribute to the research in this way.

Consideration will be given to whether it is in the participant’s interests to continue or discontinue trial treatment in the event of a serious adverse reaction. If the participant, the therapist or the research team believes that ongoing intervention or trial participation will result in, or is likely to result in, a further or ongoing serious adverse reaction, discontinuation will be recommended. Should an unexpected serious adverse reaction occur to either the therapy or the trial procedures, and if this is judged to be directly related to trial participation or to the therapy, the trial will be temporarily halted pending investigation and analysis of the extent to which future risk can be mitigated. If it is judged that this is not possible, the trial will be discontinued. This process will be led by the sponsor in collaboration with the TSC chair and chief investigator. The same process will be followed should information come to light that indicates that the therapy intervention or trial procedures are unsafe.

#### Strategies to improve adherence to interventions {11c}

Individual interviews at the beginning of the MBCT treatment phase will serve to reinforce the rationale of the research, highlight the importance of practice and address potential barriers to engagement. Participants allocated to continue with treatment as usual will take part in an interview that will serve to reinforce their understanding of the importance of their contribution to the research and prevent tendencies towards ‘resentful demoralisation’. We will offer support to help patients to familiarise themselves with the technical aspects of videoconferencing.

#### Relevant concomitant care permitted or prohibited during the trial {11d}

Patients who are currently taking antidepressant medication will be eligible for the trial and medication use will be documented for statistical analysis. All patients will be encouraged to continue treatments as usual.

#### Provisions for post-trial care {30}

Patients’ GP and referring IAPT service will be informed of trial participation and the end of trial participation in writing.

### Outcomes {12}

#### Primary outcome

The primary clinical outcome will be reductions in depression symptomatology as assessed using the PHQ-9 [20]. The primary timepoint for outcome measures will be 34 weeks post-randomisation. Hence, the primary outcome will be PHQ-9 scores at 34-week follow-up (consistent with end-of-treatment monitoring in IAPT). The PHQ-9 is a widely used self-report measure of depression that represents an integral part of the management of depression in the IAPT pathway and has good psychometric properties.

#### Secondary outcomes

Secondary outcomes include PHQ-9 measured at 10 weeks post-randomisation, and other clinical outcomes measured at 10-week and 34-week follow-up. The authors report a test–retest reliability of 0.84 over a period of 48 h [20]. Other clinical secondary outcome measures will include the Generalized Anxiety Disorder Questionnaire (GAD-7) [24], the Phobia Scale and the Work and Social Adjustment Scale, all from the IAPT minimum data set (IAPT Toolkit, 2008/9), along with the Warwick-Edinburgh Mental Wellbeing Scale (WEMWBS) [25], Experiences Questionnaire (EQ) Decentering Scale [26] and Five Factor Mindfulness Questionnaire (FFMQ) [27]. A series of binarised outcomes will be derived, based on PHQ-9 and/or GAD-7. Recovery, reliable recovery, and reliable improvement will be reported using (i) PHQ-9 only to align with depression research literature and (ii) both PHQ-9 and GAD-7, to align with IAPT practice. We will also report deterioration and reliable deterioration with regard to PHQ-9 and GAD-7 separately. We will also track symptoms weekly using the PHQ-9, GAD-7, Phobia Scale and the Work and Social Adjustment Scale.

#### Baseline survey

Participant characteristics assessed as part of the MINI interview will allow us to make comparisons between eligible patients who declined to participate, and those patients who participated in the trial.

#### Economic evaluation

The economic evaluation will take a health and social care perspective, as required for evidence presented to NICE. In addition, the cost perspective will be broadened to include the costs of time off productivity losses, since these are known to be relevant and important in those attending IAPT services [28].

Costs will be calculated by collecting service use information using the Adult Service Use Schedule (AD-SUS), a self-report measure developed by the team at King’s College and used in previous trials of MBCT [19], modified for use online, to which routine unit costs will be applied [29]. We will collect data on all service use not just use related to mental health conditions, because there is evidence that successful treatment in IAPT can reduce use of all healthcare services [30]. In addition, comparison via randomised groups will ensure that any differences in cost are due to the impact of the MBCT intervention. Information on TAU will be collected via the AD-SUS, modified to ensure that all relevant services are included. Data on the use of the MBCT intervention will be collected via therapist records and costs estimated using the standard approach set out by Curtis [29], acknowledging the challenges of costing group-based interventions [31]. Outcomes for the economic evaluation will be QALYs, calculated using health utilities derived from the EQ-5D-5L [32, 33]. Costs and outcomes will be combined first in a cost-utility analysis using QALYS and second in a cost-effectiveness analysis using the PHQ-9, providing information on whether or not MBCT is worthwhile in terms of cost savings elsewhere or improvements in outcomes, and information will be provided to decision makers with statistical analysis of differences in costs, cost-effectiveness planes and cost-effectiveness acceptability curves [34].

#### Qualitative analyses

Qualitative analyses will be used to explore patient experience of the intervention and to understand how the treatment might best benefit patients in the IAPT pathway. Previous trials have shown considerable variation in the degree to which patients engage in mindfulness practice [13] and a major focus of the qualitative analyses will therefore be on factors influencing such engagement and its relation with dynamics of change. For this purpose, we will investigate the following: (1) patients’ views on acceptability of MBCT and mindfulness practice, and the experience of participating in the course remotely; (2) patients’ views of the changes they experience and their utilisation of mindfulness skills; and (3) patients’ views of the broader impact of MBCT on their lives.

A subsample of participants in the MBCT arm, estimated to be 24 (or until data saturation has been reached), will be invited to a qualitative telephone interview conducted by trained research assistants. Recruitment will be purposive, including patients across all sites, and seeking to achieve maximum variation in relation to (1) completion/non-completion of treatment, (2) response/non-response to treatment and (3) recruitment site (to examine contextual factors).

Written feedback provided in the protocol sheets that MBCT participants receive on a weekly basis will be used to inform subsampling and will also provide us with the opportunity to explore any unanticipated experiences and effects in more depth. In collaboration with service users, we will develop, and pilot test, a semi-structured topic guide based on the above aims. Interviews will be video-recorded, transcribed verbatim, and anonymised. Thematic analysis of interview transcripts will be conducted using a Framework approach [35], involving the coding and sorting of textual units according to both deductive and inductively derived categories, and the use of matrices to review the coded data, investigate commonalities and differences and search for patterns. Coding and data management will be facilitated by NVivo software.

Given that MBCT was originally developed for relapse prevention in patients who are in remission and its use for currently depressed treatment non-responders will require adaptations in how the manualised approach is delivered and made accessible for patients, we will also conduct qualitative interviews with the therapists involved in the trial. These will serve to investigate (1) the overall experience of the therapist, (2) their experience of supporting participants in their practice, (3) helpful and unhelpful aspects of MBCT for treatment non-responders and (4) therapists’ experience of its overall effectiveness. Interviews will be guided by a semi-structured topic guide and data processed using the same approach that will be applied to participants’ interviews (see above).

### Participant timeline {13}

**Fig. 1:**
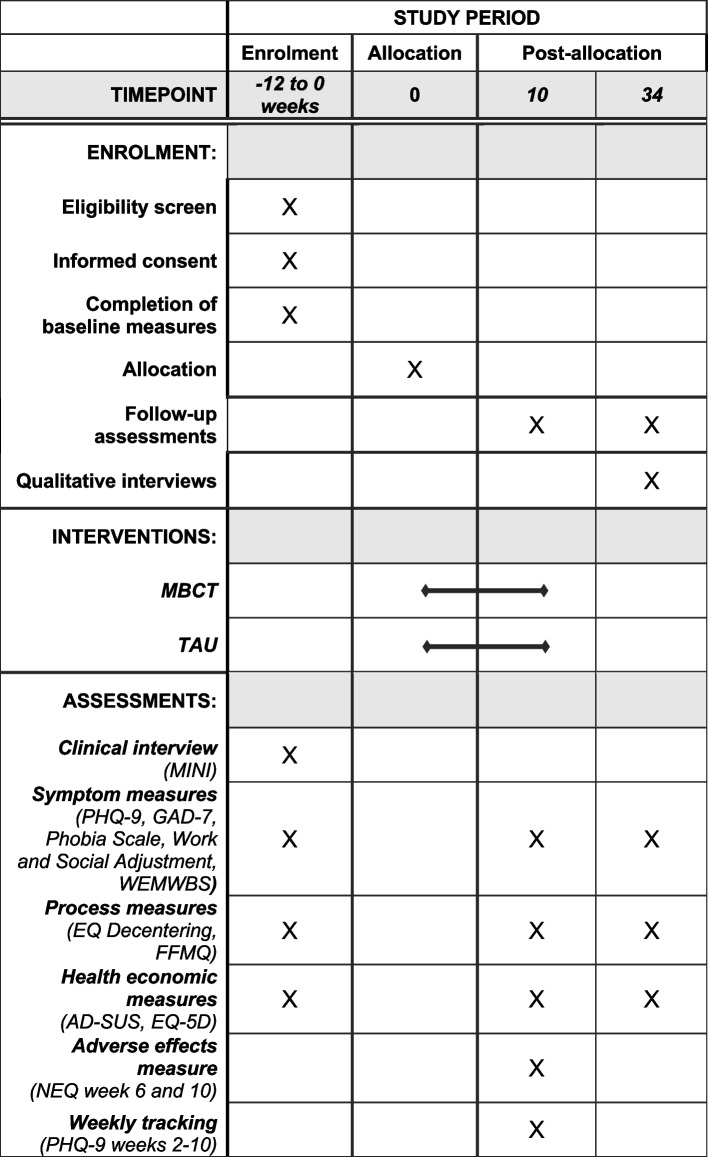
Schedule of enrolment, interventions and assessments (displayed according to Standard Protocol Items: Recommendations for Interventional Trials [SPIRIT] template)

Interested participants will undergo screening using a brief structured telephone interview conducted by the research assistant. Potential participants will be invited for an initial assessment session to be conducted via videoconferencing to ascertain eligibility using structured clinical interviews conducted by the research assistant and assess baseline levels of symptoms (baseline assessment). Eligibility interviews will be conducted as soon as possible after the screening. Baseline questionnaire assessments will be conducted within a window of 3 weeks before randomisation. Participants will be randomly allocated and learn about group assignment at least a week preceding the pre-intervention interviews held with both groups. After the 9-week treatment delivery period, participants will be assessed again at 10 weeks and 34 weeks post-randomisation. Patients will be asked to complete the follow-up assessments within a 1-week window and prompted weekly using phone, text, or email for up to four weeks, if not responsive. Patients are free to receive their usual care through the NHS while they wait to start MBCT. See Fig. 1 for a schematic diagram depicting the schedule of enrolment, interventions and assessments.

### Sample size {14}

Following previous suggestions for defining successful treatment outcome in depression [36], the study will be powered to enable detection of a Minimal Clinically Important Difference (MCID) [37]. Using a criterion of one standard error of measurement (38), the MCID for our primary outcome measure (PHQ-9) has been estimated to range from 2.59 under best-case reliability scenarios to 4.78 under worst-case reliability scenarios [38]. In order to detect an MCID at the smaller end of this range (2.59) using a standard deviation of 5.4 (as reported for the baseline data in the clinical trial that served to estimate the above-listed range of MCIDs in [39]), with 90% power at an alpha level of 0.05, 186 participants are required. In our previous large-scale multi-centre trial of MBCT for patients with a history of recurrent suicidal depression, 93% of participants provided follow-up data over a 1-year follow-up period [18]. Considering a rate of attrition of 20%, conservatively estimated to be above that observed in our previous research, we will recruit a total sample of 234 participants (117 in each arm, 78 per site). As currently available research suggests that trials using remote delivery generally show comparable or even lower rates of attrition, we would expect this estimate to be transferable to the use of videoconferencing delivery [40]. The research team will monitor attrition at regular milestones during the trial (e.g. at the point where 50% of participants have reached their scheduled 6-month follow-up time) and consider remedial steps to increase sample size, if this is needed. We have not inflated the sample size to take account of clustering within treatment groups, as reanalyses of previous trials of MBCT have found intra-class correlations (ICCs) for primary outcomes to be negligible [41].

### Recruitment {15}

Participating IAPT services will identify patients who are coming to the end of their high-intensity treatment and have not responded (PHQ-9 ≥ 10) or patients who within a 6-month window following the end of high-intensity therapy show levels of symptoms above caseness without prior remission. Data from the IAPT services originally listed as collaborators in our grant proposal indicated that we would be recruiting from a pool of more than 7500 non-responders per year. Remote delivery of the intervention will allow us to reach an even wider potential pool of participants and allow inclusion of further IAPT services given that participation in the treatment will not be restricted to people within the geographical regions of the sites. The research team will include further IAPT services as patient identification centres (PIC) to work together with the three research sites. As IAPT services can differ widely in their characteristics and organisational features of IAPT services have been shown to explain considerable variance in their outcomes (Clark et al., 2018), we will only include services that can provide a considerable number of participants and make sure that collaborating services do not show outlier characteristics. As a general rule, services included as PIC sites will have to have recovery rates above 45% and their percentage of IAPT therapists should be at least 40%. A short participant information sheet together with a ‘Permission for Researcher to Contact Form’ will be sent to potential participants either via email or post (in the latter case together with a stamped addressed envelope for their response) through the service. Potential participants will be invited to either contact the research team directly or send the completed ‘Permission for Researcher to Contact Form’ so that the researchers can contact the potential participant. If potential participants do not return the form within 14 days, they will be contacted via email, telephone or text message by service administrators, IAPT staff or Research Network Clinical Studies Officers to check whether they have received the letter and asking them if they wish to participate in the trial. In all cases, we will follow procedures that are in line with the policy of the respective trust as covered in the GDPR statement signed by each patient and will respect any opt outs that the trust may have received via national or other routes.

**Fig. 2:**
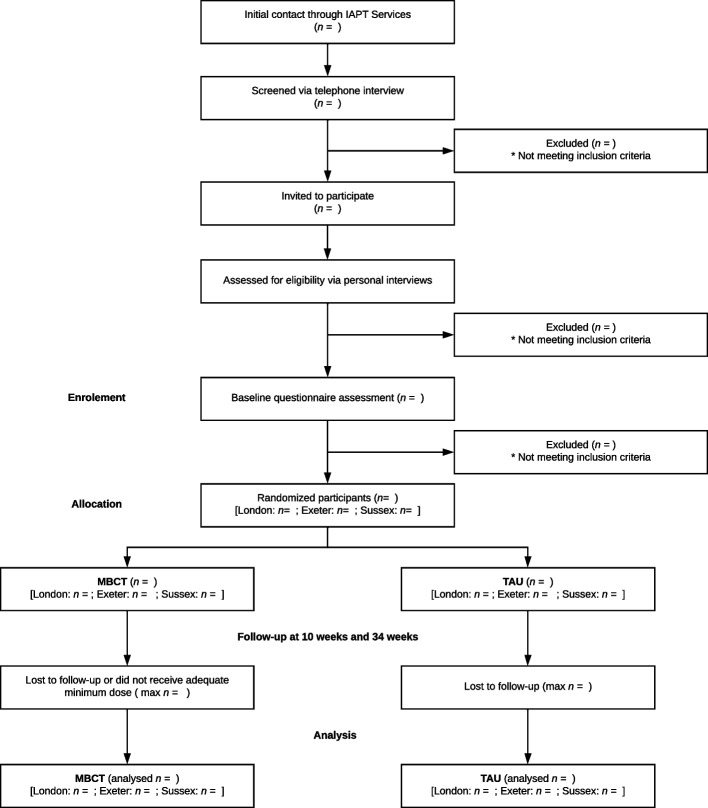
CONSORT diagram describing flow of participants through the study

Patients who are potentially interested in taking part will be contacted by the researchers for an initial screening to confirm the presence of symptoms and history of high-intensity treatment for depression, and to provide further information on the research. If positive on the screen, potential participants will be invited to take part in a structured clinical interview conducted via videoconferencing to confirm eligibility and, if eligible, to complete baseline assessments by completing web-based questionnaires. Clinical interviews will be conducted as soon as possible after initial contact and screening, while baseline questionnaire assessments will take place within a window of 4 weeks before randomisation. The invitation for this assessment will include the patient information sheet (PIS) and informed consent will be taken before the start of the assessment by asking participants to sign and return the consent form electronically. Eligible, fully informed and consenting participants will then be entered into the study and randomisation (see Fig. 2).

## Assignment of interventions: allocation

### Sequence generation {16a}

We will allocate individual participants to either MBCT or TAU at a ratio of 1:1 through remote randomisation at the UKCRC-registered Exeter Clinical Trials Unit (ExeCTU), following informed consent, completion of baseline assessment and enrolment in the trial. Randomisation will use minimisation on depression severity (PHQ-9 < 19 versus ≥ 19), antidepressant use at baseline and recruitment site. Use of a validated password website will ensure concealment. Participants will be informed of their allocation by an unblinded member of the research team.

### Concealment mechanism {16b}

Use of a validated password website will ensure concealment.

### Implementation {16c}

Participants will be informed of their allocation by a member of the research team.

## Assignment of interventions: blinding

### Who will be blinded {17a}

As baseline assessment of participants is carried out prior to randomisation, there is no risk of disclosure of treatment allocation to the assessor at the time. Use of remote assessments, initiated through automated email, will rule out any potential effects of assessors on assessments of outcomes at 10-week and 34-week follow-ups. The statistician analysing outcome data will remain blind to treatment allocation throughout the analysis, which will be conducted with groups indicated by an anonymised code. The senior statistician will be unblinded throughout.

### Procedure for unblinding if needed {17b}

In the unlikely event that a participant has an adverse reaction within either treatment arm, unblinding may be necessary. We will unblind researchers only when knowledge of the treatment arm is deemed essential to the management of the patient by their GP. Any unblinding will be recorded, although we do not expect any biasing influences on follow-up assessments given that these are conducted remotely and without direct contact with the researchers.

## Data collection and management

### Plans for assessment and collection of outcomes {18a}

At baseline, trained research assistants will administer clinical interviews via videoconferencing and ask participants to complete web-based self-report questionnaires via secure online portal. We will use the Mini-International Neuropsychiatric Interview (MINI) [21] to assess eligibility. Post-treatment and follow-up assessments will consist of questionnaires only and in line with procedures at baseline will be conducted remotely by asking participants to complete self-report questionnaires on a dedicated webpage via secure online portal. Self-report questionnaires will include the Patient Health Questionnaire 9 (PHQ-9, [20]) to assess the severity of depressive symptoms, the Generalized Anxiety Disorder Questionnaire 7 (GAD-7, [24]) to assess severity of anxiety symptoms [24], the Phobia Scale to assess symptoms of phobia, the Work and Social Adjustment Scale to assess general levels of adjustment, the Warwick-Edinburgh Mental Wellbeing Scale (WEMWBS, [25]) to assess emotional well-being, the Experiences Questionnaire Decentering Scale [26] and the Five Factor Mindfulness Questionnaire [27] to assess candidate processes of action. Health economic analyses will use the EQ-5D [32] as a generic measure of health status and a self-report version of the Adults Service Use Schedule (AD-SUS) to assess health service use [19].

### Plans to promote participant retention and complete follow-up {18b}

Use of remote assessments will reduce burden on participants and serve to promote retention.

### Data management {19}

Randomisation, data management and quality assurance will be undertaken by ExeCTU under the supervision of the CI, senior trial statistician and quality assurance manager. Routine clinical notes will be stored according to standard practice within the NHS services hosting the research. Recordings of the videoconferencing therapy sessions along with the automatically produced transcripts of the sessions will be stored on a secure server at the University of Surrey where they will be accessible to the lead scientists for purposes of therapist supervision and manual adherence checks. Data from the assessments will be entered by the research team on a secure, web-based system maintained by the ExeCTU. Data from online questionnaires will be quality checked by the research team. Consent forms will be stored separately from data and data will be anonymised wherever possible.

The datasets generated during and/or analysed during the trial will be stored in a non-publicly available repository at Sussex Partnership NHS Foundation Trust upon publication of main study results. Anonymised data may be accessed and analysed by members of the project team and by researchers collaborating with members of the project team on the analysis of these data. All personal identifiable data, with the exception of the consent form and the video recordings, will be destroyed as soon as the study closes, unless participants have consented to be contacted for future research, in which case we will keep their contact details for 5 years. Audio recordings of qualitative interviews will be destroyed immediately after transcription. Research data with personal information removed and replaced through a code and original research records, including video recordings of assessment interviews and therapy sessions, will be retained for 10 years, before being destroyed. The electronic records will be kept for 10 years after the end of the study. Publications will not contain any patient-identifiable information.

### Confidentiality {27}

Any information collected as part of the trial will be kept strictly confidential within the research team and the services involved. Both within the research team and the services confidentiality will be broken only in exceptional circumstances, if it is felt by the researcher or therapist that a patient or someone else is at immediate risk and the team will need to contact GPs or other relevant professionals.

All data will be stored and processed in line with General Data Protection Regulation (GDPR, 2018). Personal data will be link-anonymised and identified by a code known only to the research team. Names and contact details will be stored in password-protected files on secure servers and separately to link-anonymised data. In order to assess manual adherence and therapist competency, therapy sessions will be video recorded with the consent of all participants appearing in the recording. Access to these recordings and the transcripts of the sessions will be restricted to the research team and collaborating researchers. Recordings and anonymised transcripts will be stored on secure servers at the University of Surrey.

Direct quotations from qualitative interviews may be used; however, it will not be possible to identify the participant from these. Clinical records will be stored on secure servers with access restricted to the trial manager and clinical team.

### Plans for collection, laboratory evaluation and storage of biological specimens for genetic or molecular analysis in this trial/future use {33}

As described under 26b, there will be no biological specimens collected.

## Statistical methods

### Statistical methods for primary and secondary outcomes {20a}

All analyses will be carried out using an a priori statistical analysis plan as agreed with the TMG and TSC.

Participant characteristics at baseline (including number of previous depressive episodes and IAPT service) will be set out descriptively by the treatment arm. The primary analysis approach will use the intention-to-treat (ITT) principle (all participants will be included in the analysis according to their randomised allocation irrespective of the treatment actually received) including observed data only. All outcomes will be reported descriptively at baseline, and at 10 and 34 weeks’ follow-up. Continuous outcomes will be analysed using linear regression models. The binary outcome variables will be analysed using logistic regression. All analyses will adjust for participant covariates used in randomisation, with adjustment for baseline scores for continuous outcomes. We will assess other participant characteristics at baseline and will consider adjusting for any covariates that are found to be substantively unbalanced, should such covariates be considered predictive of outcome. Inferential between-group comparisons (MBCT vs TAU) for the primary and all secondary outcomes will be performed at 34-week follow-up. As a sensitivity analysis, we will perform a complier average causal effect (CACE) analysis for continuous outcomes only, to estimate the treatment effect while accounting for non-adherence to treatment. A participant in the intervention arm will be considered to be a ‘complier’, if a minimum of four treatment sessions were attended. Mixed effects regression models with a random effect on individual participant will be performed for continuous and binary outcomes, including participants with outcome data reported for at least one follow-up time. To address the potential for clustering effects by IAPT service, we will perform sensitivity analyses using mixed effects regression models for the primary and secondary (continuous and binary) outcomes, with a random effect on IAPT service. Similarly, to address the potential for effects of clustering by therapist, further sensitivity analyses will test for the effect of individual therapists or therapist seniority. Mixed effects model with a random effect on individual therapist will also be performed. As a further sensitivity analysis, therapist seniority will be added as a fixed effect within the primary model for the primary outcome and all secondary outcomes. To explore effects under conditions of different inclusion criteria, a sensitivity analysis will be run including only those patients who failed to meet both criteria for reliable recovery according to IAPT conventions, that is reliable change in symptoms and symptom levels below caseness. We will also perform a sensitivity analysis (primary outcome only) to include any data collected outside the 7-day window. Sensitivity analyses will be based on the ITT principle (except the CACE analysis). Should further sensitivity analyses be indicated, these will be described in the statistical analysis plan.

### Interim analyses {21b}

A Data Monitoring and Ethics Committee will look at outcome data regularly during data collection.

### Methods for additional analyses (e.g. subgroup analyses) {20b}

We do not a priori plan to conduct subgroup analyses.

### Methods in analysis to handle protocol non-adherence and any statistical methods to handle missing data {20c}

To investigate the effects of missing data, a further sensitivity analysis will use multiple imputation to impute missing outcome data for continuous outcomes only.

### Plans to give access to the full protocol, participant-level data and statistical code {31c}

Data sharing will be enabled using a controlled access model in line with Good Practice Principles for Sharing Individual Participant Data from Publicly Funded Clinical Trials from the UK Medical Research Council [42]. Scientists seeking to access the data for use in future projects must do so via request to the CI and projects using the data must have been approved in accordance with contemporary UK ethical and regulatory processes pertaining to the release of anonymised data under these circumstances. We will follow current recommendations on anonymising and curating trial data for sharing [43].

## Oversight and monitoring

### Composition of the coordinating centre and trial steering committee {5d}

The CI will assume responsibility for the overall management of the trial and delivery of the work. The CI will lead the core research team (including all site leads, the trial manager, and research assistants), who will meet monthly via videoconference and receive input from the wider research group and representatives of the Patient Advisory Group at quarterly Trial Management Group meetings. The Trial Management Group will monitor all aspects of the conduct and progress of the trial, ensure that the protocol is adhered to and take appropriate action to safeguard participants and the quality of the trial.

The trial is governed by a Trial Steering Committee (TSC), which is independent from the sponsor. The role of the TSC, which includes an independent chair and four other independent members, one of whom is an independent patient and public involvement representative, is to provide critical scrutiny to the conduct of the proposed research. Prof David Clark (University of Oxford) has kindly agreed to chair the TSC.

### Composition of the data monitoring committee, its role and reporting structure {21a}

We have set up an Independent Data Monitoring Committee (IDMC) comprising a chair (Prof Dean McMillan, University of York), an independent mental health statistician and a clinician. The IDMC will review serious adverse events that are thought to be trial- or treatment-related and look at outcome data regularly during data collection. As the TSC, the IDMC is independent from the sponsor and has no competing interests. The TSC and IDMC will meet on a half-yearly basis.

### Adverse event reporting and harms {22}

#### Risk monitoring

In order to identify risk issues, research assistants will screen questionnaires within 72 h of completion to check for increases in suicidality and any service use that may be indicative of a serious adverse event. A score of more than 0 on the PHQ-9 item 9 (that represents a change from the previous trial assessment) and reports of suicidal ideation, intent, plans or urges, and any risk of harm to self or others in the MINI interview or other contexts will be deemed as risk issues. Identification of a risk issue will trigger the trial team to capture more detailed information and context to assess risk in line with the trial risk protocol. Where necessary participants will be provided with support in line with the local sites’ risk management process.

In the MBCT arm, the site RA will routinely monitor PHQ-9 scores on a weekly basis prior to each session (or as soon after as possible in the event of participant non-completion) and immediately email the mindfulness teacher and PI if:

- A participant’s score has increased by 6 points or more from baseline assessment, specifying the amount of increase, and/or
- A participant scores 1 or more on item 9 of PHQ-9, specifying what the score is and whether this score is typical or represents a change

The mindfulness teacher or appropriate clinical delegate will follow the study risk protocol to ensure appropriate contact is made with the participant to discuss their mental state, current risk and what is needed to keep themselves safe. Information from this conversation will be considered by the therapist and PI to answer the question of whether a participant should continue with treatment. All instances in which the risk protocol has been enacted will be documented using the Risk Assessment Form and logged in the study risk management log together with contextual information and their classification.

#### Adverse event and serious adverse event recording and reporting

Adverse events and reactions will be defined as follows.

##### Adverse event (AE)

Any untoward medical occurrence in a patient treated on a study protocol, which does not necessarily have a causal relationship with a study intervention. An AE can therefore be any unfavourable and unintended sign, symptom or disease temporally associated with the use of the study intervention, whether or not related to that study treatment.

##### Adverse reaction (AR)

All untoward and unintended responses related to a study intervention. A causal relationship between a study intervention and an adverse event is at least a reasonable possibility, i.e. the relationship cannot be ruled out as there is evidence or arguments to suggest a causal relationship.

##### Unexpected adverse reaction (UAR)

An adverse reaction, the nature or severity of which is not consistent with the information about the trial intervention.

Serious adverse event (SAE) or serious adverse reaction (SAR) or suspected unexpected serious adverse reaction (SUSAR): Respectively any adverse event, adverse reaction or unexpected adverse reaction that:

- Results in death
- Is life-threatening (where the term life-threatening refers to an event in which the patient is at risk of death at the time of the event; it does not refer to an event that might hypothetically cause death if it was more severe (e.g. a silent myocardial infarction))
- Requires inpatient hospitalisation or prolongation of existing hospitalisation
- Results in persistent or significant disability or incapacity
- Consists of a congenital anomaly or birth defect
- Or any other health event which in the opinion of the clinician is serious

Important adverse events that are not immediately life-threatening or do not result in death or hospitalisation but may jeopardise the subject or may require intervention to prevent one of the other outcomes listed in the definition above should also be considered serious.

As suicidal ideation and mild self-harm are a common aspect of the clinical presentation of many mood disorders, we will only rate these as adverse events if they include suicidal behaviour or the degree of self-harm puts the individual at risk of physical injury. In particular, suicidality will be deemed as an AE or SAE, if risk is categorised as level C on our risk protocol. Routine hospitalisations and planned surgery recorded at the baseline assessment visit (pre-treatment) do not require reporting as SAEs.

Any event observed by either a researcher or therapist that could be considered as SAE will be documented on the Serious Adverse Event Form and reported to the local PI. Research Assistants will be responsible for screening the AD-SUS questionnaire within 72 h of completion to check for potential serious adverse events. Identification of a potential SAE in the AD-SUS will trigger a telephone call to the participant to capture more detailed information and context of the event although this should not delay reporting. As reports on the AD-SUS are retrospective, the research team will assess any remaining risks and the local sites risk management process will be adhered to at any time to ensure participants receive support where necessary.

The local PI will evaluate the reported event for seriousness considering the available contextual information. All non-serious AE will be documented in the electronic patient record. If the issue is assessed as serious, the event must be reported to the CI, Trial Manager, local R&D department and Sponsor, immediately and no later than 24 h of being made aware of the event. Initial reports of SAE can be made via email but must be promptly followed with a detailed written report using the Serious Adverse Event Reporting Form for the Sponsor and containing sufficient detail regarding concurrent life events. The PI should ensure that follow-up information is provided when available.

The sponsor will allocate an SAE number and forward the event to the trial’s Independent Clinician who is part of the Independent Data Monitoring Committee. The Independent Clinician will rate the event to confirm or question its seriousness, with the final decision in case of discrepancy made by the PI; determine the causality of SAEs according to Table 1; and also rate SAEs with regard to its expectedness. Independent review will be conducted within 72 h and the outcome will be reported back to the local Research Assistant PI, CI, Trial Manager and sponsor by email using the SAE number allocated by the sponsor.

**Table 1:** SAE causality rating

Relationship	Description	Event type
Unrelated	There is no evidence of any causal relationship	Unrelated SAE
Unlikely to be related	There is little evidence to suggest that there is a causal relationship (e.g. the event did not occur within a reasonable time after administration of the trial treatment). There is another reasonable explanation for the event (e.g. the participant’s clinical condition or other concomitant treatment)	Unrelated SAE
Possibly related	There is some evidence to suggest a causal relationship (e.g. because the event occurs within a reasonable time after administration of the trial treatment). However, the influence of other factors may have contributed to the event (e.g. the participant’s clinical condition or other concomitant treatment)	SAR
Probably related	There is evidence to suggest a causal relationship and the influence of other factors is unlikely	SAR
Definitely related	There is clear evidence to suggest a causal relationship and other possible contributing factors can be ruled out	SAR

**Fig. 3:**
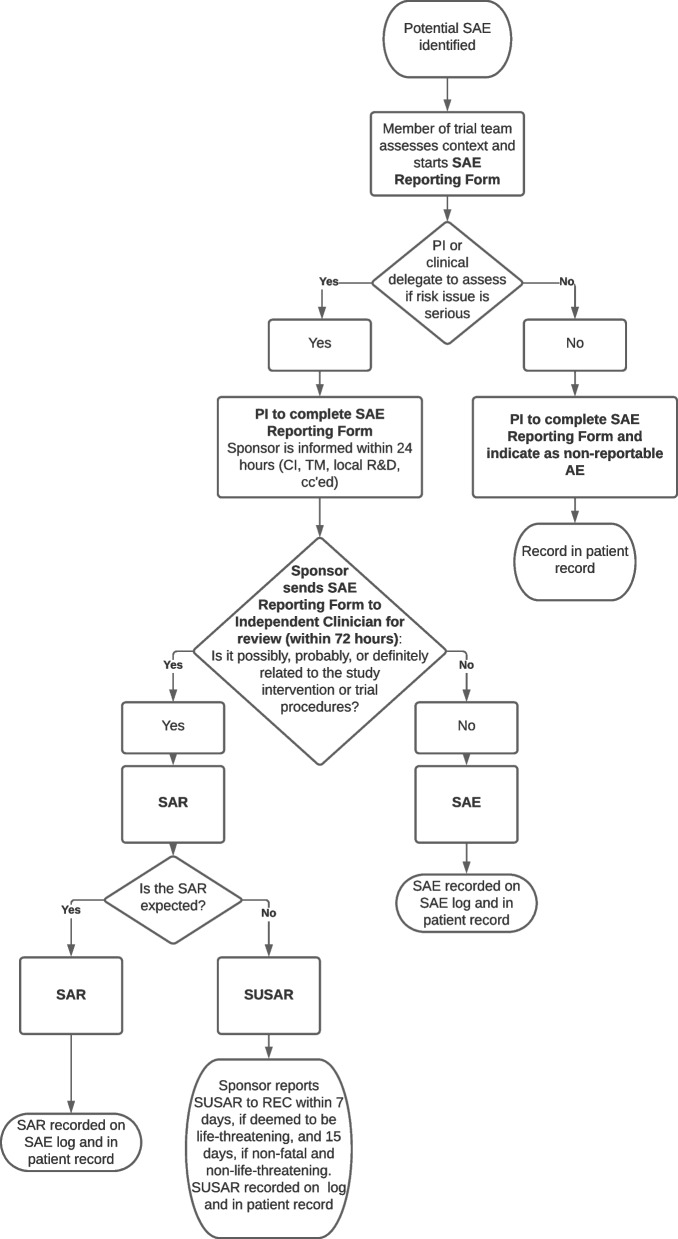
SAE reporting flowchart

SAEs classed as related and unexpected will be reported to the Research Ethics Committee by the sponsor within 7 days if it is deemed to be life-threatening or results in death and 15 days if it is non-fatal and non-life threatening (see Fig. 3).

The PIs will take responsibility for making sure that the local risk management procedures are adhered to at all times and that all risk issues are followed up until resolution.

### Frequency and plans for auditing trial conduct {23}

The research will be audited through established procedures at the sponsor’s R&D department.

### Plans for communicating important protocol amendments to relevant parties (e.g. trial participants, ethical committees) {25}

In case amendments to the protocol are needed, we will seek to obtain sponsor approval for the amendment to be submitted. We will prepare a submission to the REC through the IRAS system (https://www.myresearchproject.org.uk/help/hlpamendments.aspx#Amendment-Tool), authorised by the CI and the sponsor. The CI will communicate the outcome of the review process and any resulting changes of the protocol to the sites and inform participating organisations. Trial registrations and the published protocol will be amended accordingly.

## Dissemination plans {31a}

The trial data may have the potential to inform changes in current practice within IAPT. The evaluated treatment manual of MBCT for patients with current symptoms of depression will facilitate training and dissemination of the approach within IAPT and other contexts. Insights from qualitative analyses will provide information on implementability. The findings of the research will be disseminated using the widest range possible of peer-reviewed scientific journals and professional publications. We will present results at conferences and workshops and disseminate findings through media and social media where possible. We will also disseminate findings on a local level, to participants, services and other stakeholders.

## Discussion

This trial will provide definitive evidence on whether the use of MBCT in depressed IAPT high-intensity treatment non-responders is clinically effective and cost-effective. The evidence from this trial is intended to inform changes in current practice within IAPT. Should MBCT for IAPT treatment non-responders prove clinically effective and cost-effective, the data from this study together with accumulated evidence from previous research on MBCT for depressed treatment non-responders would justify changes in service provision that will have significant impact on the mental and physical health of the large number of patients who have come to the end of the IAPT pathway but have not recovered sufficiently.

## Trial status

The project started on the 1st of January 2021 and sites have been initiated for recruitment in April and May 2021 (Exeter: 20/04/2021; Sussex and London: 26/04/2021). The study database was signed off on 18/05/2021 and first consents were obtained on 9/06/2021 in Exeter, 22/05/2021 in London and 01/07/2021 in Sussex. We expect recruitment to be completed in the middle of October 2022. Should targets for the current cohorts not be reached, recruitment will remain open into the first quarter of 2023.

## Supplementary Information

**Additional file 1.** Consent form.

## Data Availability

The CI will serve as the custodian of the trial data. There are no contractual agreements in place that would limit access for the investigators.

## Abbreviations

AD-SUS: Adult Service Use Schedule
AE: Adverse event
AR: Adverse reaction
CI: Chief investigator
CTQ: Childhood Trauma Questionnaire
CTU: Clinical Trials Unit
DMEC: Data Monitoring and Ethics Committee
DSM-5: Diagnostic and Statistical Manual of Mental Disorders, Fifth Edition
EQ: Experiences Questionnaire
EQ-5D: EuroQoL 5-Dimension instrument
FFMQ: Five Factor Mindfulness Questionnaire
GAD-7: Generalized Anxiety Disorders Questionnaire-7
GP: General Practitioner
HRA: Health Research Authority
IAPT: Increasing Access to Psychological Therapies
ICC: Intra-class correlation
IRAS: Integrated Research Application System
ISRCTN: International Standard Randomised Controlled Trial Number
MCID: Minimal clinically important difference
MBCT: Mindfulness-Based Cognitive Therapy
MBI-TAC: Mindfulness-Based Interventions – Teaching Assessment Criteria
NEQ: Negative Effects Questionnaire
NHS: National Health Service
NICE: National Institute for Clinical Excellence
NIHR: National Institute for Health Research
PHQ-9: Patient Health Questionnaire 9
PIS: Patient information sheet
QALY: Quality-adjusted life year
RCT: Randomised controlled trial
RfPB: Research for Patient Benefit
SAE: Serious adverse event
SAR: Serious adverse reaction
SCID: Structured Clinical Interview for DSM-5
SUSAR: Suspected unexpected serious adverse reaction
TAU: Treatment as usual
TSC: Trial Steering Committee
UAR: Unexpected adverse reaction
UK: United Kingdom
WEMWEBS: Warwick-Edinburgh Mental Wellbeing Scale (WEMWBS)
