# Supplementary material for: A randomised controlled trial to investigate the clinical effectiveness and cost effectiveness of Mindfulness-Based Cognitive Therapy (MBCT) for depressed non-responders to Increasing Access to Psychological Therapies (IAPT) high-intensity therapies: study protocol
Source: Trials. 2023 Jan 19;24:43. doi: 10.1186/s13063-022-06882-w (PMC9851098; doi:10.1186/s13063-022-06882-w)
Supplement: Supplementary file 1 — Additional file 1. Consent form. [file 13063_2022_6882_MOESM1_ESM.pdf]

TRUST LOGO

Add contact details of the local research team and Local Investigator

Study Code: *Site ID Code:*

Participant identification number:

CONSENT FORM

Title of Project: **A Research Study Comparing the Effects of Mindfulness-Based Cognitive Therapy and Care as Usual in Patients who are Continuing to Suffer from Depression after IAPT High-Intensity Therapy**

Name of Researcher: ______________________________ *If you agree, please initial box*

1. I confirm that I have read the information sheet dated................... (version............) for this study. I have had the opportunity to consider the information, ask questions and have had these answered satisfactorily.	
2. I understand that my participation is voluntary and that I am free to withdraw at any time without giving any reason, without my medical care or legal rights being affected.	
3. I understand that the information held and maintained by ______________________________ *(IAPT service)* may be used to help contact me or provide information about my health status and that the research team will provide the service with information about my participation in the study.	
4. I agree to the therapy and assessment sessions being audio and video recorded and transcribed.	
5. I agree to my General Practitioner being informed of my participation in the study.	
6. I consent to the processing of my personal information for the purposes explained to me. I understand that such information will be handled in accordance with the terms of the General Data Protection Regulation.	
7. I agree for study data to be shared with my IAPT service in case I am allocated to receive Mindfulness-Based Cognitive Therapy.	

8. I agree to the use of anonymised quotes from the interview about my experience of the intervention in case I am among the participants who take part in this interview.	
9. I agree to take part in this study.	

Additional:		
	Yes	No
10. I agree to be contacted about ethically approved research studies for which I may be suitable. I understand that agreeing to be contacted does not oblige me to participate in any further studies.		
	Yes	No
11. I agree for my anonymised data, anonymised transcripts and session recordings to be used in future research, which has ethics approval in accordance with contemporary UK ethical and regulatory processes, by researchers of good standing, within 10 years after the end of this study, here or abroad. Any use of data in further research will be in line with the Good Practice Principles for Sharing Individual Participant Data from Publicly Funded Trials from the UK Medical Research Council.		
12. I would like to receive a summary of the results of this research once it is finished.		

________________________ __________________ ____________________________

Name of Participant *Date* *Signature*

________________________ __________________ ____________________________

Name of Person taking Consent *Date* *Signature*

**1 copy for participant; 1 copy for researcher site file; 1 (original) to be kept in medical notes* (if participant is a patient).
